# Catechin reduces phototoxic effects induced by protoporphyrin IX-based photodynamic therapy in the chick embryo chorioallantoic membrane

**DOI:** 10.1117/1.JBO.25.6.063807

**Published:** 2020-02-12

**Authors:** Jaroslava Joniová, Georges Wagnières

**Affiliations:** Swiss Federal Institute of Technology (EPFL), Institute of Physics, Laboratory for Functional and Metabolic Imaging, Lausanne, Switzerland

**Keywords:** catechin, reactive oxygen species, protoporphyrin IX, photodynamic therapy, phototoxic effect, photoprotection, chorioallantoic membrane, photobleaching

## Abstract

**Significance:** Side effects of many cancer treatments are associated with the production of reactive oxygen species (ROS) in normal tissues. This explains why patients treated by photodynamic therapy (PDT) often suffer from skin photosensitization, whereas those subject to radiotherapies frequently experience damages in various organs, including the skin.

**Aim:** Catechin, which belongs to the natural flavanols family, is well known for its antioxidant properties. Hence, our main objective was to investigate whether catechin can reduce damages induced by PDT using protoporphyrin IX (PpIX-PDT), an endogenous photosensitizer commonly used in dermatology.

**Approach:** An *in vivo* model, the chick embryo chorioallantoic membrane (CAM), was used for this study. An amount of 20  μl of a solution containing 5-aminolevulinic acid, a natural precursor of PpIX, was applied topically on the CAM 4 h before PDTs (405 nm, $2.9\;\mathrm{mW}/{\mathrm{cm}}^{2}$, $1.2\;J/{\mathrm{cm}}^{2}$). Catechin was applied at different concentrations (1 to 50  μM) and times (0 to 240 min) before PDT. In addition, we assessed the potency of catechin to reduce the PpIX fluorescence photobleaching induced by PDT.

**Results:** We observed that catechin significantly reduces the vascular damages generated by PpIX-PDT. Moreover, we have shown that catechin inhibits PpIX photobleaching.

**Conclusions:** These observations suggest that catechin significantly reduces the level of ROS produced by PpIX-PDT.

## 1 Introduction

Photodynamic therapy (PDT), a minimally invasive and clinically approved procedure, is used for the treatment of different types of (pre)cancers as well as certain forms of age-related macular degeneration.^1^^,^^2^ Three key elements are involved in PDT: a nontoxic photosensitizer (PS), light that excites the PS, and molecular oxygen. The PS, after excitation with light, can transfer some of its energy to the latter, which leads to the production of reactive oxygen species (ROS), in particular, singlet oxygen. These ROS induce cell/tissue damages, eventually resulting in cell death by apoptosis, necrosis, and/or autophagy.^3^^–^^5^

One of the most extensively used PS for PDT is protoporphyrin IX (PpIX). This fluorescing PS is ubiquitously present and endogenously produced in most living cells as a penultimate step in the biosynthesis of heme.^6^ Its production in certain cancer lesions can be significantly increased by the administration of some of its approved natural precursors, 5-aminolevulinic acid (5-ALA), or derivatives thereof. However, normal tissue inflammation, erythema, edema, pain, and/or itching have been reported as side effects of PDT.^7^ One of them results from the skin photosensitizations induced by sunlight when patients are treated with most of the approved PSs. These negative effects, which develop in the course of, or immediately after (hours/days) PDT, are frequently linked to the generation of ROS, especially singlet oxygen, in the normal/healthy tissues.^8^^,^^9^

**Fig. 1:**
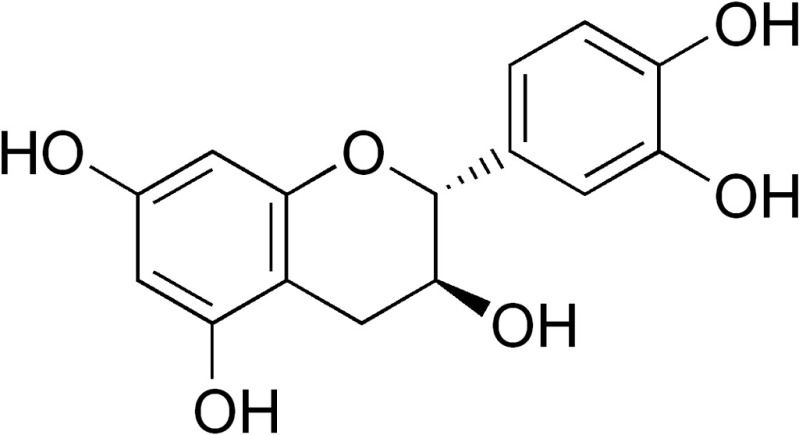
Chemical structure of catechin.

Therefore, in this study, we have investigated the ability of catechin (Fig. 1), a natural antioxidant belonging to the family of flavonoids (polyphenols), to reduce the phototoxic effects of ROS induced by PpIX-PDT.

Flavonoids are an extensive group of polyphenolic antioxidants naturally occurring in fruits, vegetables, cocoa, chocolate, and several beverages such as wine and green tea.^10^^–^^12^ More precisely, catechin belongs to the family of flavanols (or flavan-3-ols), which are able to act as ROS scavengers, especially superoxide anions and singlet oxygen, thus preventing the production of free radicals.^13^^,^^14^

Furthermore, catechins also induce indirect antioxidant mechanisms, including the inhibition of pro-oxidant enzymes, the induction of antioxidant enzymes, and many others.^15^

The chicken embryo chorioallantoic membrane (CAM) has been used as an *in vivo* model in this study to quantify the photoprotective effects of catechin when vascular damages were induced by PpIX-PDT.

The CAM is widely used as a relevant model for studying angiogenic agents due to its developing vascular network. Indeed, the CAM plays the same role as the placenta in mammals, by supplying the nutrition and oxygen to the developing chicken embryo.^16^ During the embryo development, several days after the beginning of the incubation, the CAM is formed with tree-like venules and arterioles that evolve from the initial homogeneous capillary plexus.^17^ Its accessibility and the two-dimensional structure of the vascular network make the CAM an excellent and very convenient model for the visualization of the vascular network and, in particular, to assess the effects of PDT.

In this study, different concentrations of catechin were topically administered at different times before PpIX-PDT.

## 2 Material and Methods

### 2.1 Chemicals

All chemicals were purchased from Sigma-Aldrich, Switzerland.

### 2.2 CAM

Fertilized chicken eggs were purchased from Animalco AG, Switzerland. For 3 days, eggs were incubated at 37°C in a humidified and automatically rotating incubator with the blunt end up. At the embryo development day (EDD) 3, a small hole (diameter: 3 mm) was made at the pointed end of the shell and covered with tape. Afterward, eggs were placed back to the incubator in a stationary position until further use. On EDD 12, the hole was enlarged (diameter: 25 mm) enabling topical ALA and catechin administration followed by PDT.

### 2.3 PpIX-PDT

PpIX-based PDT has been described in detail in our previous reports.^18^ Briefly, ALA was dissolved in an aqueous solution of NaCl (0.9% of NaCl and 20 mg of ALA per ml with a pH of 6.8). An amount of 20  μl of this solution was topically applied on the CAM. Then, 4 h after ALA administration, PDT was conducted with the light delivered by an epifluorescent microscope (Eclipse E 600 FN Nikon) utilizing a 4× objective (Nikon, NA: 0.13, Plan Fluor, WD 17.1). PpIX produced endogenously was excited with light at 405 nm (filter cube BV-2A, Nikon, Japan). PpIX-PDT was performed with a light dose of $1.2\;J/{\mathrm{cm}}^{2}$ (irradiance: $2.9\;\mathrm{mW}/{\mathrm{cm}}^{2}$). This light dose typically induces a grade 4 vascular damage according to the PDT effects scale defined by Lange et al.,^19^ i.e., vessels with a diameter <70  μm are all closed by PDT.

### 2.4 Catechin Administration

A stock solution of catechin was dissolved in EtOH before further dilution in NaCl (used for the topical administration on the CAM). Different concentrations of catechin (1, 5, 10, 20, 30, and 50  μM) were applied topically (in 100  μl of NaCl) to the CAM at different time intervals ranging between 0 and 4 h prior to PpIX-PDT. In all experiments, the content of EtOH was <1%. It should be noted that concentrations of catechin are relative to the weight of the embryo at given EDD, i.e., 5 g at EDD 12. For all control eggs, 100  μl of NaCl (without catechin) was topically applied on the CAM.

### 2.5 Angiograms Acquisition and Analysis

Fluorescent angiograms of the CAM’s vascular network were recorded 24 h after PDT. Angiograms were obtained by an intravenous injection (20  μl) of fluorescein isothiocyanate–dextran (25 kD, 25 mg/ml) dissolved in NaCl. To further improve the contrast between the extravascular space and blood vessels of the CAM, India ink (Parker) was concurrently injected (100  μl) under the CAM. It has been proven that India ink, which is also used as a marker for tumor margination in the human skin, is not toxic to the CAMs in our conditions.^20^ Angiograms were acquired with an epifluorescent microscope (Eclipse E 600 FN Nikon) utilizing a 4× objective (Nikon, NA: 0.13, Plan Fluor, WD 17.1). PDT potency along with the assessment of the catechin photoprotection was quantified by the quantitative analysis of these fluorescent angiograms using an ImageJ Macro (NIH, Bethesda, Maryland) developed in our laboratory.^20^

### 2.6 PpIX Photobleaching Study

Fluorescent images for this substudy have been obtained with the same microscope setup as described above. A BV-2A filter cube (excitation: 405 nm; Nikon, Japan) combined with a longpass emission filter transmitting light above 610 nm (E610LP filter; Chroma, Irvine, California) was used for PpIX fluorescence visualization and relative quantification. Images were recorded at the beginning (first 5 s) and at the end (last 5 s) of the PDT irradiations. The ImageJ software was then used to quantify the PpIX fluorescence intensity changes (at the beginning and at the end of illumination) in the presence and absence of catechin. This study was carried out with a light dose of $10\;J/{\mathrm{cm}}^{2}$ at 405 nm, and 10  μM catechin was administered 15 min before PDT.

## 3 Results and Discussion

### 3.1 Study of the Photoprotective Effects of Catechin Applied at Different Concentrations

As mentioned above, PpIX is one of the most frequently used PSs for PDT. Its phototoxic effects are well documented, in particular, those induced in the vascularization.^18^^,^^21^^–^^23^ Hence, in this study, it has been used as PS for the induction of vascular damages in the CAM. On the basis of a preliminary study, we established the spectral and radiometric conditions inducing grade 4 vascular damages according to Lange et al.^19^ (data not shown). We concluded that light at 405 nm applied with a dose of $1.2\;J/{\mathrm{cm}}^{2}$ and an irradiance of $2.9\;\mathrm{mW}/{\mathrm{cm}}^{2}$ was appropriate. These conditions were used in all experiments presented in Secs. 3.1 and 3.2.

**Fig. 2:**
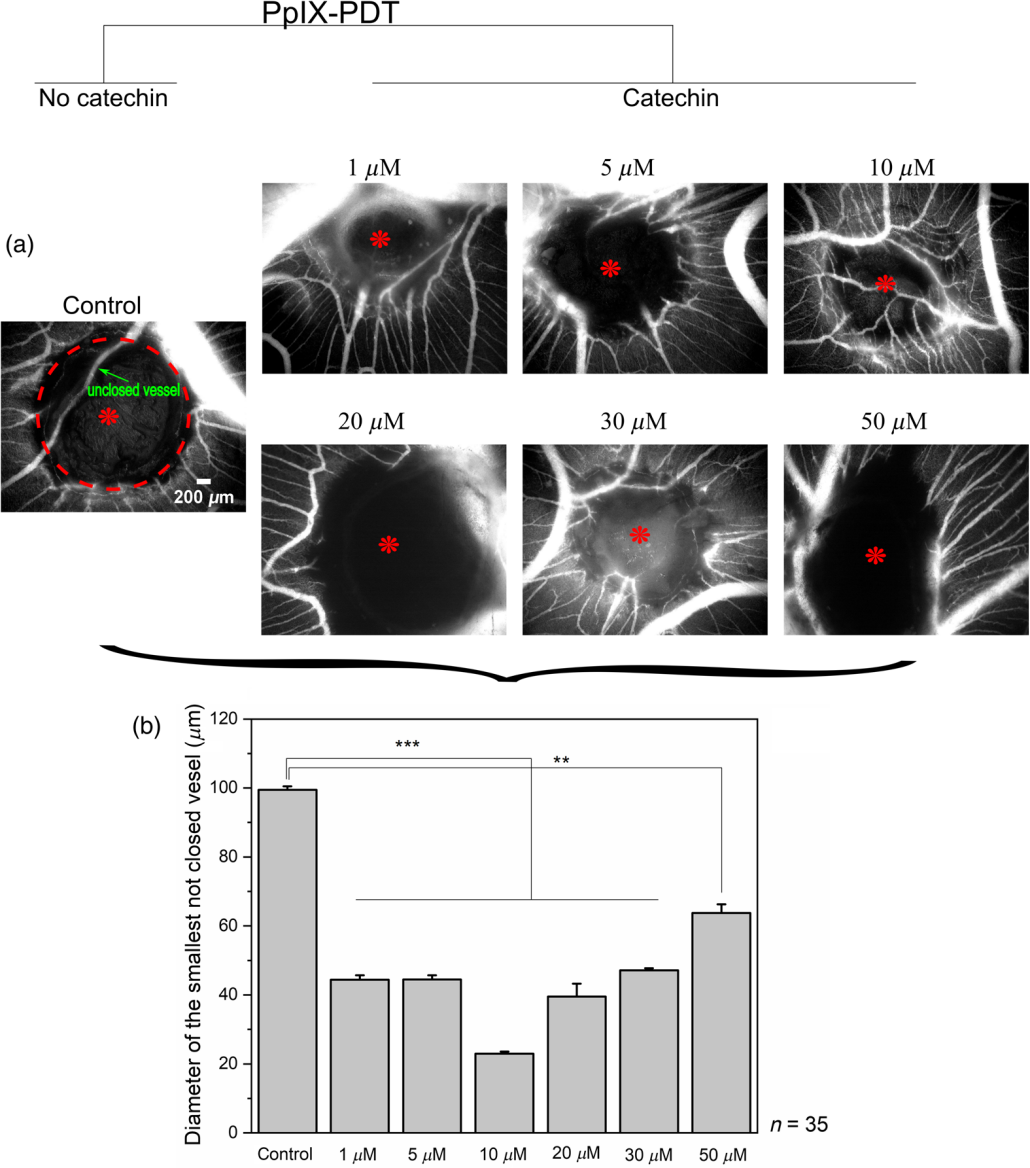
Assessment of PpIX-PDT on the CAM in the presence of different concentrations of catechin. (a) Representative FITC-dextran fluorescent angiographies of the CAM in control samples (no catechin) and in the presence of different concentrations of catechin. Images were acquired 24 h after PDT. All images have the same magnification. A typical unclosed vessel as well as the illuminated circular area is presented in the control image only for the sake of clarity. Red asterisks in the images represent the centers of the illumination with a diameter of 2 mm. (b) Quantitative analysis of the fluorescent images. Number of eggs: n=35. The error bars represent the standard error of the mean. Significant p values are represented as **p≤0.01, ***p<0.001.

In the first step, we studied the photoprotective effects of catechin applied topically at different concentrations 15 min before PDT (1 to 50  μM). Figure 2(a) shows illustrative fluorescence angiograms acquired 24 h after PDT. A quantitative analysis of fluorescence angiograms [Fig. 2(b)] to assess the efficacy of PpIX-PDT, i.e., to determine the diameter of the smallest vessels that did not close after PpIX-PDT, showed that, in the control samples, this diameter was in the order of 100  μm. On the other hand, PpIX-PDT was significantly less potent at all concentrations of catechin. More precisely, the small concentrations (1 and 5  μM) reduced the PpIX-PDT effects in such a way that the smallest vessels that did not close had an average diameter of about 45  μm. Similarly, the administration of catechin at higher concentrations (20, 30, and 50  μM) led to a reduction of the smallest not closed vessel down to 39, 47, and 64  μm, respectively. An amount of 10  μM of catechin showed the most significant photoprotective effect since the average diameter of the smallest unclosed vessels was about 23  μm. It should be noted that “no light” controls (in the presence of catechin only or a combination of catechin with ALA) were conducted, resulting in an absence of vascular changes (data not shown).

Although the family of catechins is well recognized for its antioxidant properties, it should be noted that they are also able to act as pro-oxidants generating ROS.^24^^,^^25^ Indeed, although their antioxidant properties are due to their ability to act as free radical/ROS scavengers, their presence can stimulate hemoglobin-induced protein oxidation. This could be caused by their antioxidant capability, which promptly induces an oxidative degradation of hemoglobin,^22^ an effect that may explain the concentration-dependent photoprotective properties of catechin.

### 3.2 Determination of the Optimal Catechin Administration Time Before PpIX-PDT

**Fig. 3:**
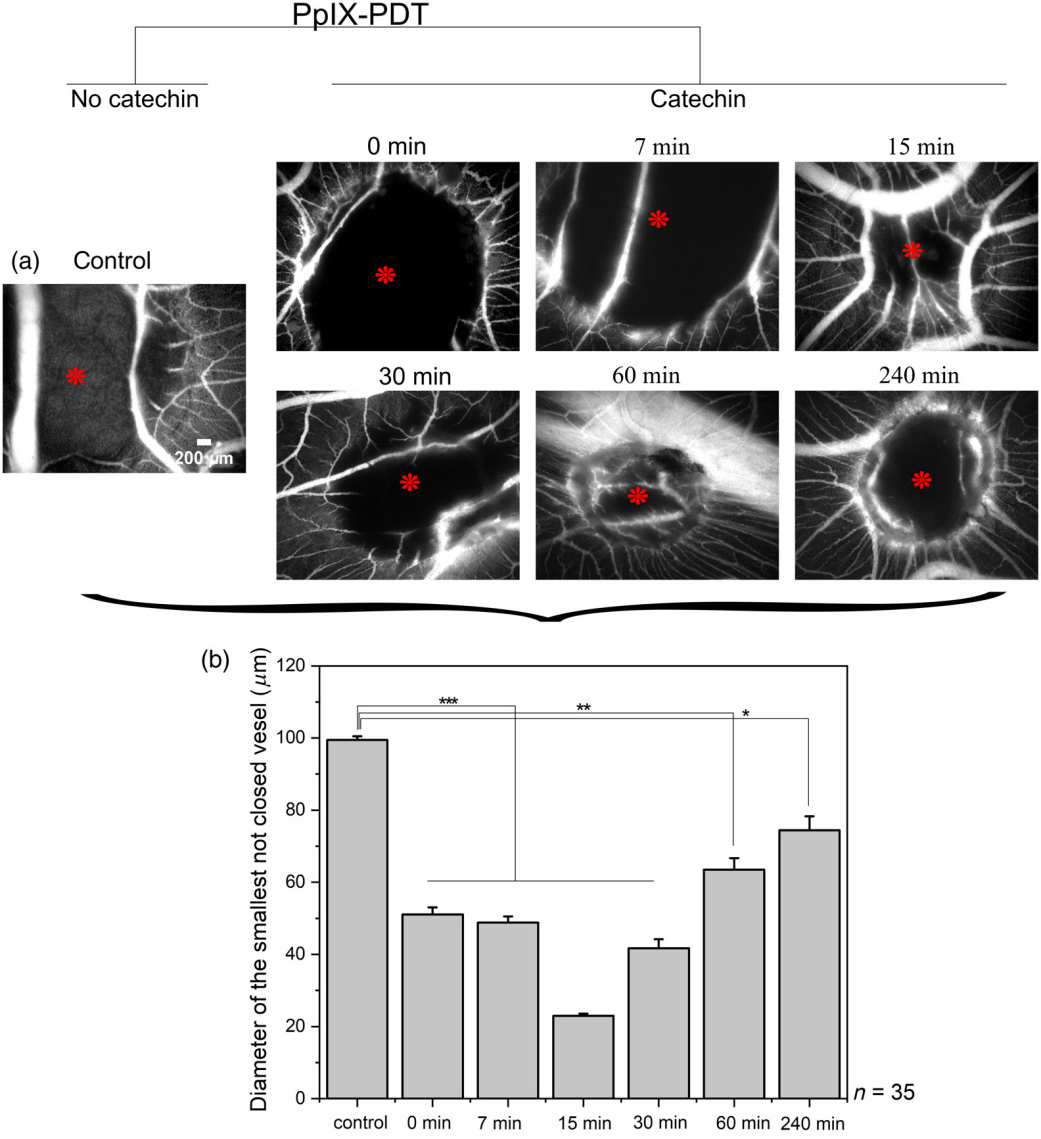
Effects of PpIX-PDT on the CAM when catechin is applied at different times before illumination. (a) Representative FITC-dextran fluorescent angiographies of the CAM in control samples (no catechin) and in the presence of 10  μM of catechin administered at different times before PpIX-PDT. Images were acquired 24 h after PDT. Red asterisks in the images represent the center of the illumination spots with a diameter of 2 mm. (b) Quantitative analysis of the fluorescent images for different times between catechin administration and PpIX-PDT. Number of eggs: n=35. The error bars represent the standard error of the mean. Significant p values are represented as *p≤0.05, **p≤0.01, ***p<0.001.

Following the catechin concentration study reported in Sec. 3.1, we investigated the influence of the role played by the time separating the catechin administration and PpIX-PDT. Since 10  μM of catechin was found to be optimal, this concentration was used for the second substudy. Figure 3 shows the vascular effects of PpIX-PDT when catechin was applied topically at different times before illumination on the CAM.

We observed that the presence of 10-μM catechin reduces the PpIX-PDT effect at all times. When administered just (i.e., <30  s) before PpIX-PDT illumination, the smallest vessel that did not close was in the order of 51  μm. Increasing the time between catechin administration and illumination up to about 15 min led to more potent inhibitions of the vascular effects of PpIX-PDT. Indeed, when catechin was applied 7 min before PDT, the average diameter of the smallest unclosed vessel was 48  μm, whereas this diameter went down to 23  μm at the optimal time of 15 min. With longer times (30 min and more), catechin became less and less potent to generate a photoprotection.

According to our knowledge, there is no literature on the catechin pharmacokinetics in the CAM. However, studies conducted on rodents, dogs, rabbits, and humans showed that, after oral administration, catechin is mainly metabolized by phase-2 conjugation process in the liver and intestine.^26^ In our study conducted on the CAM, the reduced photoprotection observed at times >30  min could be explained by the catechin internalization by the CAM and chicken embryo itself.

### 3.3 Assessment of the ROS Production During PDT Based on the Measurement of the PpIX Fluorescence Photobleaching

**Fig. 4:**
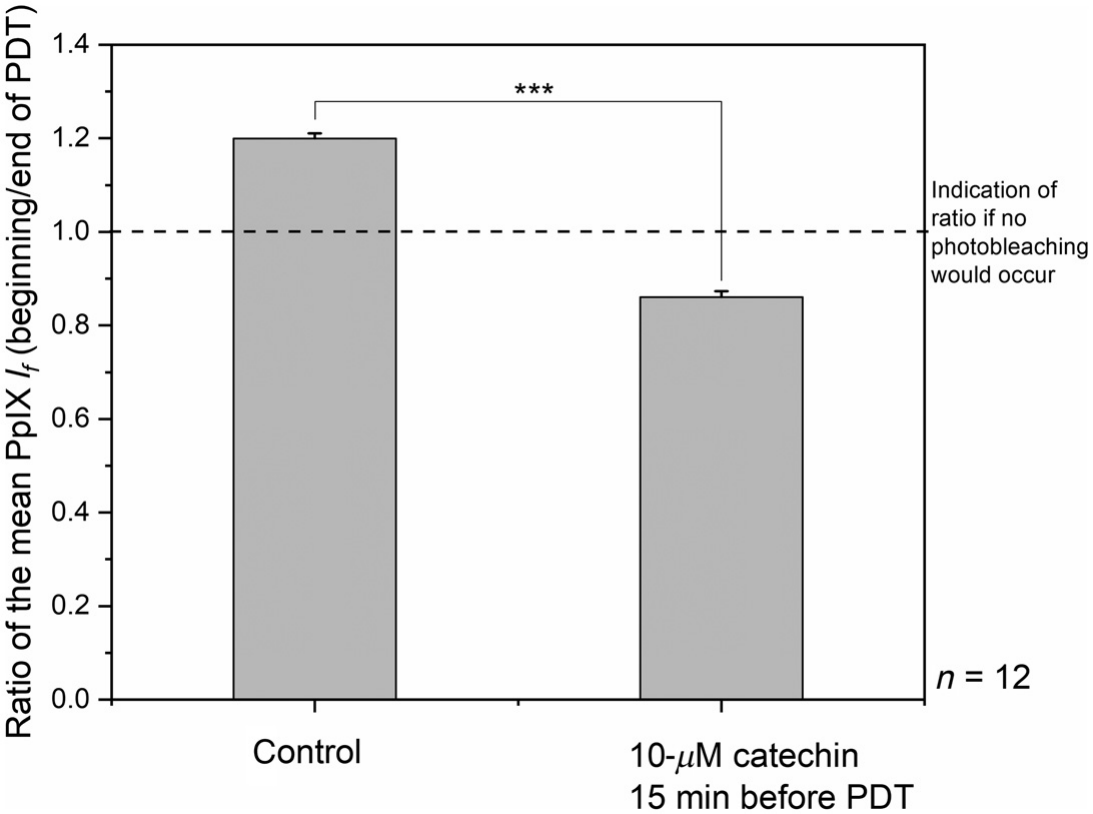
Reduction of the PpIX photobleaching with catechin. PpIX fluorescence intensity was recorded in the first 5 s and last 5 s of the PDT illumination. These values were divided and compared for the control samples and when 10-μM catechin was administered 15 min before PDT. Number of studied eggs: n=12. The error bars represent the standard error of the mean. Significant p value is represented as ***p<0.001.

Measurements conducted in this part of the study were based on the observation that PpIX photobleaches during PDT, an effect that is directly related to the ROS (mostly singlet oxygen) production.^27^ Indeed, Moan and Berg^28^ demonstrated that the more ROS are created, the more PpIX is photobleached. Therefore, it is of interest to determine if a correlation exists between the photoprotective effects of catechin and the PpIX photobleaching. Thus, this estimation of the protective effect of catechin was compared with its ability to prevent photobleaching of PpIX. Figure 4 shows the ratio of the fluorescence intensity of PpIX measured at the beginning (first 5 s) divided by this intensity measured at the end of PDT (last 5 s), both for a control group (no catechin) and in the presence of 10-μM catechin applied 15 min before PpIX-PDT. If no photobleaching would occur, the ratio of the PpIX fluorescence at the beginning and at the end of illumination would be equal to 1.

For the purpose of acquiring a good signal-to-noise ratio, the light dose used for this part of the study was increased to $10\;J/{\mathrm{cm}}^{2}$ for all conditions (controls and 10-μM catechin). It should be noted that increasing the light dose did not preclude the photoprotective effect of catechin. At the same time, it is interesting to point out that when photobleaching experiments of PpIX were conducted with $1.2\;J/{\mathrm{cm}}^{2}$ (see Secs. 3.1 and 3.2), the PpIX photobleaching was almost not measurable (data not showed), although the tissue damages were significant. On the whole, these observations suggest that only a small fraction of the PpIX present on the CAM membrane 4 h after ALA application is actually localized in compartment(s), probably the mitochondria, playing a crucial role in the photosensitization process. This suggests that the majority of the PpIX fluorescing in the CAM in our conditions plays a reduced role in the photosensitization process, if any, probably because it is outside these critical compartments.

Nevertheless, as shown in Fig. 4, 10-μM catechin not only reduces the PpIX photobleaching, but leads to an increase of the PpIX fluorescence in the CAM. This effect is probably due, at least in part, to the ability of catechin to reduce the ROS concentration. It could also be that the increase of PpIX fluorescence observed during PDT in the presence of catechin is due to a chelation of iron by this flavanol, as postulated by Samman et al.^29^ This mechanism may impair the availability of iron to convert PpIX in heme, thus leading to an increase of the PpIX fluorescence detected in the CAM.

## 4 Conclusion

It is well known that patients treated by PDT often suffer from skin photosensitization. In addition, those patients subject to radiotherapies frequently experience damages in various organs, including the skin, as a consequence of the ROS production in the tissues. Since the family of catechins is well known for its antioxidant properties, we investigated if its topical application could induce photoprotective effects in normal tissues during PpIX-PDT and/or if it could be used to protect the skin of patients against photosensitization induced by PpIX-PDT. The influence of the catechin concentration and the time between its administration and PpIX-PDT were studied on the CAM vascularization. We observed that catechin reduced the PpIX-PDT potency in all conditions we explored, whereby 10-μM catechin administered topically 15 min before PDT had the most potent photoprotective effect. In addition, we demonstrated that, in these conditions, catechin diminishes the PpIX photobleaching induced by PpIX-PDT. Although additional experiments exploring other administration conditions carried out on different model systems are necessary, our results suggest that catechin is an interesting candidate to protect normal tissues during PDT or radiotherapy.
